# Supplementary material for: Molecular Dynamics Simulations of HPr Proteins from a Thermophilic and a Mesophilic Organism: A Comparative Thermal Study
Source: Int J Mol Sci. 2023 May 31;24(11):9557. doi: 10.3390/ijms24119557 (PMC10253394; doi:10.3390/ijms24119557)
Supplement: Supplementary file 1 [file ijms-24-09557-s001.zip › ijms-2392922-supplementary.pdf]

Supplementary Materials

Molecular Dynamics Simulations of HPr Proteins from a Thermophilic and a Mesophilic Organism: A Comparative Thermal Study

AAna K. Gómez-Flores 1, Edgar López-Pérez 2 and Salomón J. Alas-Guardado 1,*

[1] Departamento de Ciencias Naturales, Universidad Autónoma Metropolitana Unidad Cuajimalpa, Mexico City 05300, Mexico

[2] Posgrado en Ciencias Naturales e Ingeniería, Universidad Autónoma Metropolitana Unidad Cuajimalpa, Mexico City 05300, Mexico

* Correspondence: salas@cua.uam.mx; Tel.: +52-55-5814-6500 (ext. 3868)

In this Supplementary Materials, we present full details of the systems of two proteins, details about the main calculations done in this work, and additional results related to the different simulated trajectories.

S1. Details about Both Systems

(a) **(b)**

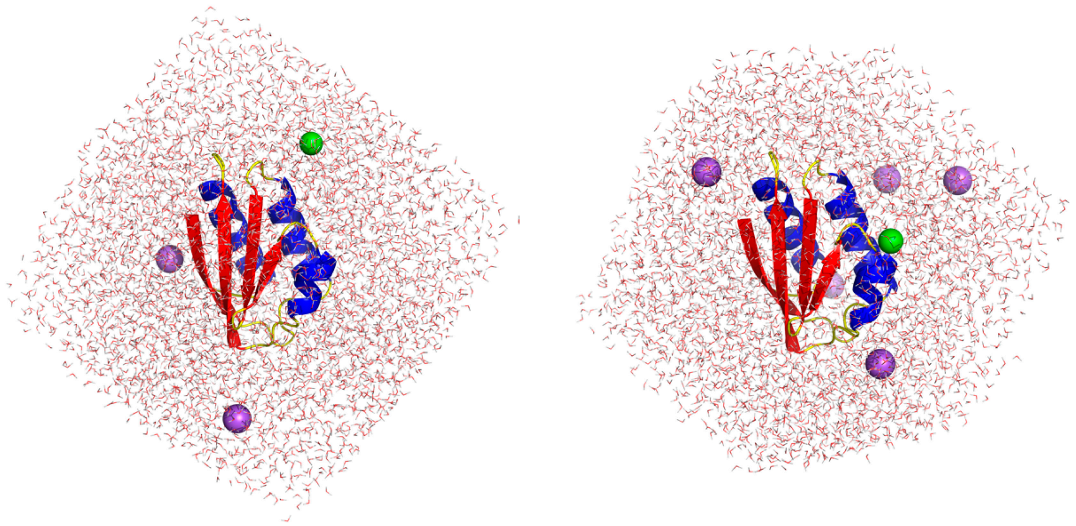

Figure S1. Snapshots of both systems at t = 0 ns of simulation at 298 K: (**a**) *Bst*HPr and (**b**) *Bs*HPr proteins. The red, blue, and yellow colors indicate the β-strand, α-helix, and random coil structures, respectively. Cl^- and Na^+ counterions are colored in green and purple circles, respectively. The rest of the particles are solvent molecules, in this case, water.

S2. Structural Parameters Calculated

S2.1. Secondary Structure Profiles

Secondary structure profiles were calculated with the *do_dssp* function of GROMACS, which compares the structural coordinates generated at each time step during the trajectory with the specifications given in the DSSP dictionary and assigns the corresponding secondary structure. The DSSP dictionary is based on the recognition of hydrogen bond patterns and geometrical features extracted from X-ray crystallographic coordinates [1].

S2.2. Root Mean Square Deviation

This parameter was determined using the *rms* function of GROMACS. The first conformation of the protein obtained during the simulation (production stage) is used as reference structure (t = 0 ns). RMSD is calculated as follows

$$\mathrm{RMSD}(t_1,t_2)=\left[\frac{1}{M}\sum_{i=1}^{N} m_i \left\| \boldsymbol{r}_i(t_1)-\boldsymbol{r}_i(t_2)\right\|^2\right]^{\frac{1}{2}} \tag{S1}$$

where N is the number of atoms, m_i is the mass of the atom i, $\boldsymbol{r}_i(t)$ is the position vector for the atom i at time t, and M is the total mass.

S2.3. Radius of Gyration

This indicator was measured employing the *rg* function of GROMACS. Rg is calculated from the center of mass of the protein and follows the next equation

$$\mathrm{Rg}=\left(\frac{\sum_i \left\|\boldsymbol{r}_i^2\right\| m_i}{\sum_i m_i}\right)^{\frac{1}{2}} \tag{S2}$$

where m_i is the mass of atom i and $\boldsymbol{r}_i$ is the position vector of atom i with respect to the center of mass.

S2.4. Native Contacts

The fraction of native contacts was calculated by mean of the MDtraj program [2]. This program considers the model of Best *et al.* [3], and it is given by

$$\mathrm{Q}(t)=\frac{1}{N}\sum_{i,j}\frac{1}{1+\exp\left[\beta\left(r_{ij}(t)-\lambda r_{ij}^{N}\right)\right]} \tag{S3}$$

where Q(t) is the fraction of native contacts calculated for each protein conformation at time t, $r_{ij}(t)$ is the distance between atoms i and j at time t, r_{ij}^{N} is the distance between heavy atoms i and j in the native state, N is the set of total pairs heavy atoms (i,j) belonging to residues θ_i and θ_j so that $|\theta_i - \theta_j| > 3$ Å and $r_{ij}^{N} < 4.5$ Å, β is a smoothing parameter (β = 5 Å^{-1}), and λ is a fluctuation factor (λ = 1.8).

S3. Molecular Interactions Calculated

S3.1. Hydrogen Bonds

This parameter was calculated using the *hbond* function of GROMACS. Its calculation considers the distance r and angle α between the mass center of the acceptor (A) and donor (D) atoms of protons (H), which are $r_{AD} \leq 3.5$ Å and $\alpha_{AD} \leq 30^{o}$ [4]. Moreover, in this work, we evaluated two HBs types: a) HB present in the interior of the protein structures (labeled as HBpp) and b) those formed between the protein and solvent (labeled as HBps).

S3.2. Solvent Accessible Surface Area

The hydrophobic contacts were indirectly evaluated through the parameter SASA. In this work, we used the freeSASA program [5] to obtain the polar and nonpolar areas of the residues exposed to the solvent. This program considers the Lee and Richards's approximation (1.4 Å solvent radius) [6].

S3.3. Salt Bridges

The GetContacts tool [https://getcontacts.github.io] was used to determine the frequency of salt bridges during the trajectories. It was taken as a criterion that one SB is stable when it has a frequency of 0.30, i.e., two oppositely charged residues that interact 30% during the simulation are considered stable salt bridges. In addition, the time evolution of the distance r_{SB} between ion pairs is calculated using the *mindist* function of GROMACS. Both measurements are based on the criterion of Barlow and Thornton [7], in which $r_{SB} \leq 4$ Å. Moreover, we calculated intra- and intermolecular salt bridge types.

S4. Electrostatic Surface Potential

The electrostatic surface potential was obtained from the Poisson-Boltzmann equation. The calculation of the ESP was performed with the Adaptive Poisson-Boltzmann Solver (APBS) software [8,9] using a target grid resolution of 0.5 Å. ESP visualization is observed when projected onto the solvent-accessible surface of the proteins using a spherical solvent probe with a radius of 0.9 Å. Calculations and visualization were carried out with the APBS plugin of PyMOL.

S5. Structural Analyses

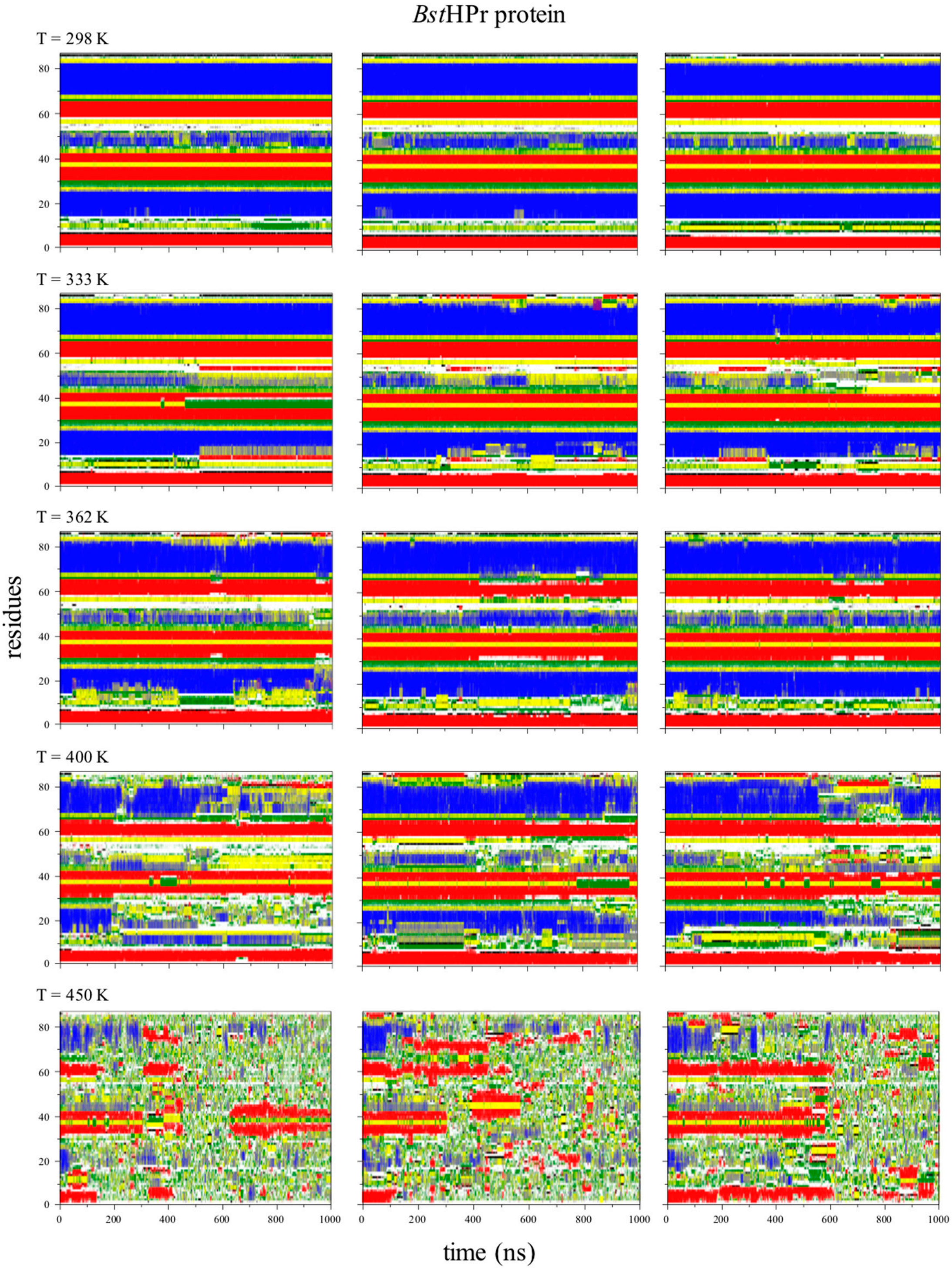

Figure S2. Secondary structure behavior of *Bst*HPr for the three simulations at different temperatures studied in this work. The columns from left to right correspond to simulations 1, 2, and 3. The color code of the structures is: random coil (white), β-sheet (red), β-bridge (black), bend (green), turn (yellow), α-helix (blue), π-helix (purple), and 3_{10}-helix (gray).

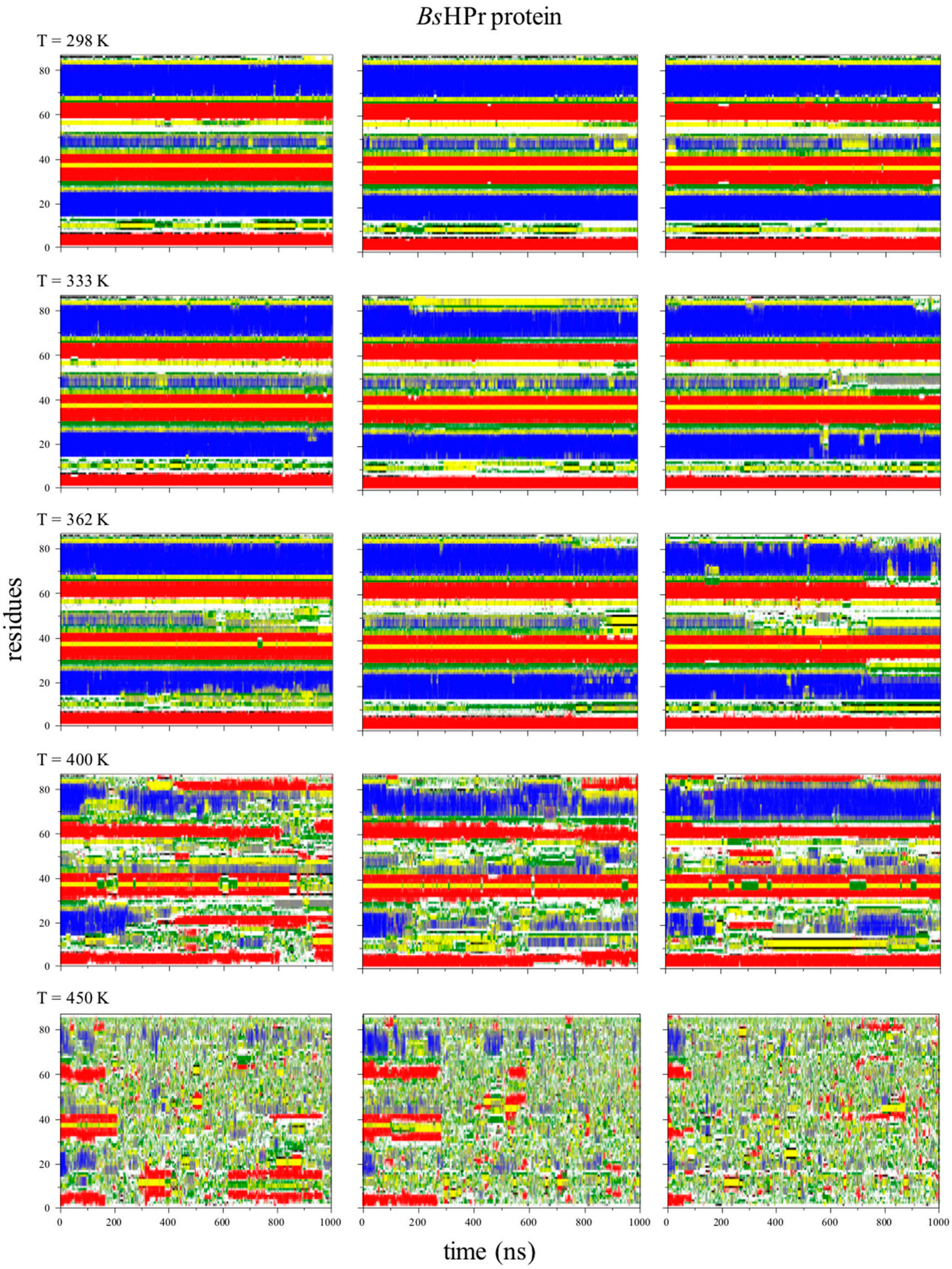

Figure S3. Secondary structure behavior of *Bs*HPr for the three simulations at different temperatures studied in this work. The columns from left to right correspond to simulations 1, 2, and 3. The color code of the structures is: random coil (white), β-sheet (red), β-bridge (black), bend (green), turn (yellow), α-helix (blue), π-helix (purple), and 3_{10}-helix (gray).

Figure S4a shows the Ramachandran plot of the α_2-helix structure of the thermophilic protein at 298 K for simulation 1. Although most of the residues are located in the region that allows the conformation of this structure, there is a clear dispersion towards other less favorable regions. In addition to the α_2-helix region, the formation of two other regions belonging to the coil and left-handed helix conformations are observed. In the case of the mesophilic protein, similar behavior is observed, but with lower dispersion towards less favorable areas both in the region of the α_2-helix structure and in the non-permitted regions (Figure S4b). This occurs because the α_2-helix structure contains a Gly49 residue in the two proteins. As the Gly residue has only one hydrogen atom for its side chain, i.e., the glycine lacks the methylene group at Cβ, it can populate different areas of the Ramachandran diagram without steric constraints, giving greater conformational freedom. In addition, glycine residues are known to destabilize α-helix structures, favoring the unfolding of them. Therefore, these facts can explain the instability of the α_2-helix structure from the native state (298 K), since it is lost in some time span during the MD simulations for both proteins.

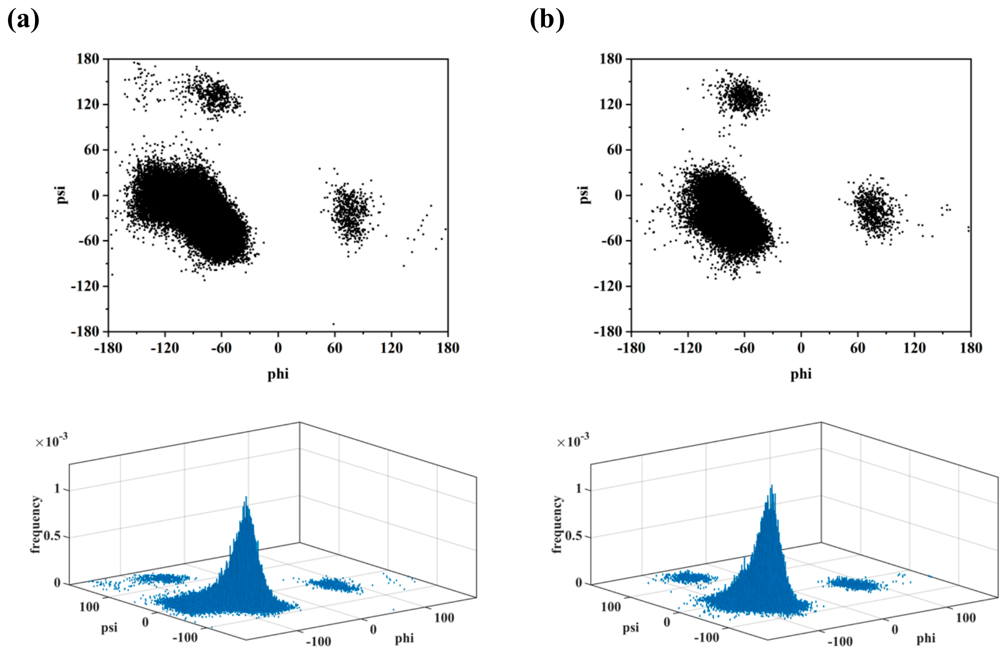

Figure S4. (top panels) Ramachandran plots of the α_2-helix structure for: (**a**) *Bst*HPr and (**b**) *Bs*HPr proteins at 298 K. The bottom panels show the corresponding frequency diagrams.

Consequently, by increasing the temperature at 333 K it is expected that the α_2-helix structure will increase its dispersion even more than at 298 K (Figure S5), favoring its unfolding much below the *Bst*HPr melting temperature. This can be clearly observed at different times of the three simulations at 333 K for both proteins (see Figures S2 and S3).

In the same context, Figure S6 shows the Ramachandran diagrams of the α_3-helix structure for the two proteins at 298 K, which do not present dispersions as high as those observed in the α_2-helix structure. In this case, the dihedral angles of the α_3-helix are located

in the most favorable regions of the conformation of the α-helix structure. Note that the α_3-helix structure of the thermophilic protein is less dispersed than that of the mesophilic protein.

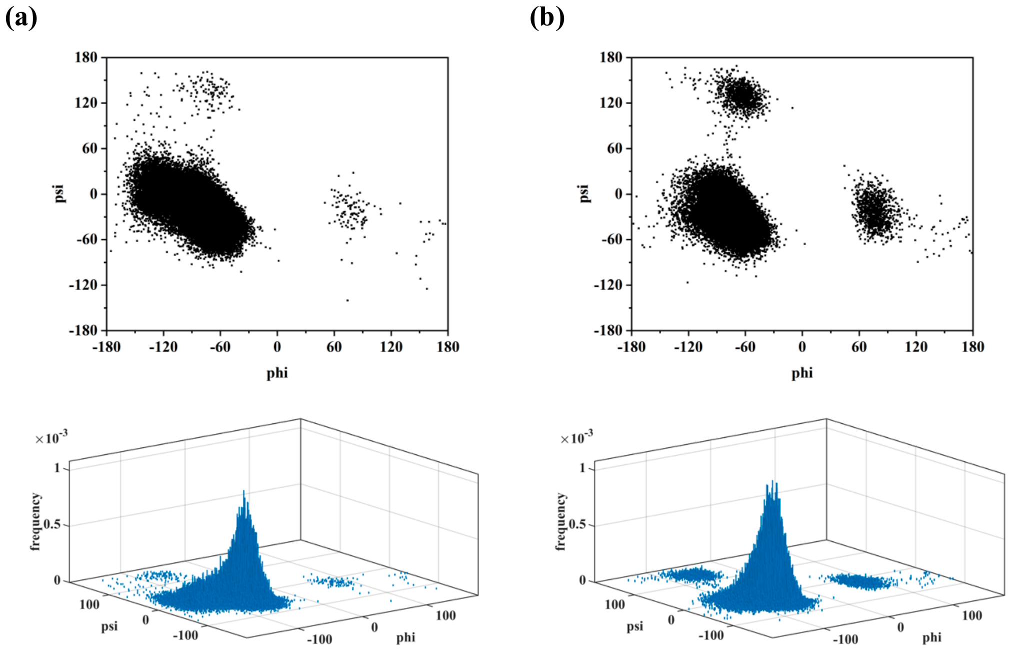

Figure S5. (top panels) Ramachandran plots of the α_2-helix structure for: (**a**) *Bst*HPr and (**b**) *Bs*HPr proteins at 333 K. The bottom panels show the corresponding frequency diagrams.

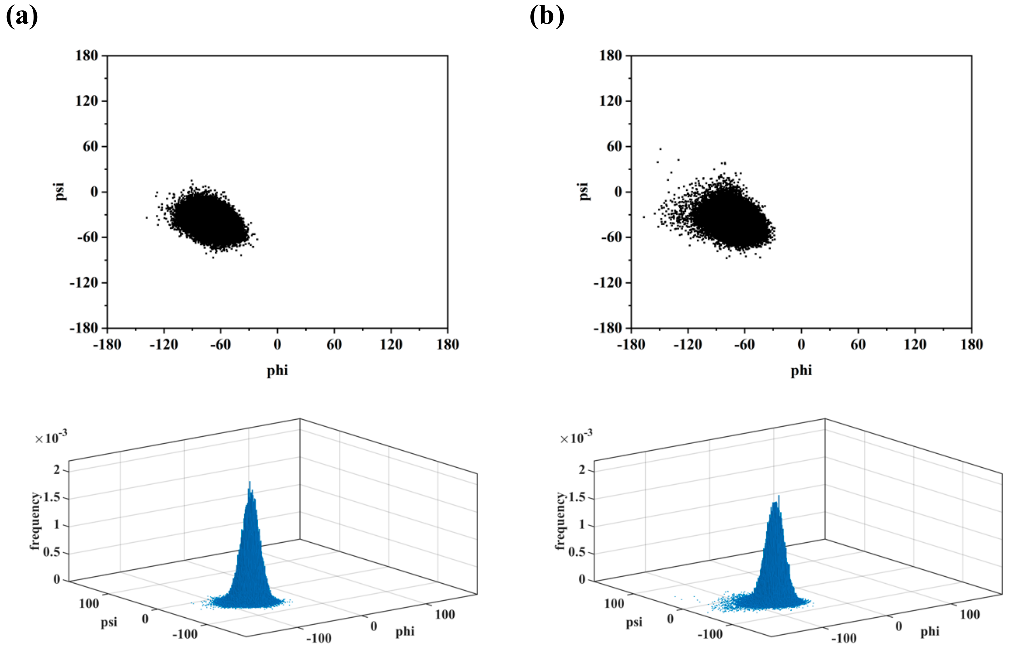

Figure S6. (top panels) Ramachandran plots of the α_3-helix structure for: (**a**) *Bst*HPr and (**b**) *Bs*HPr proteins at 298 K. The bottom panels show the corresponding frequency diagrams.

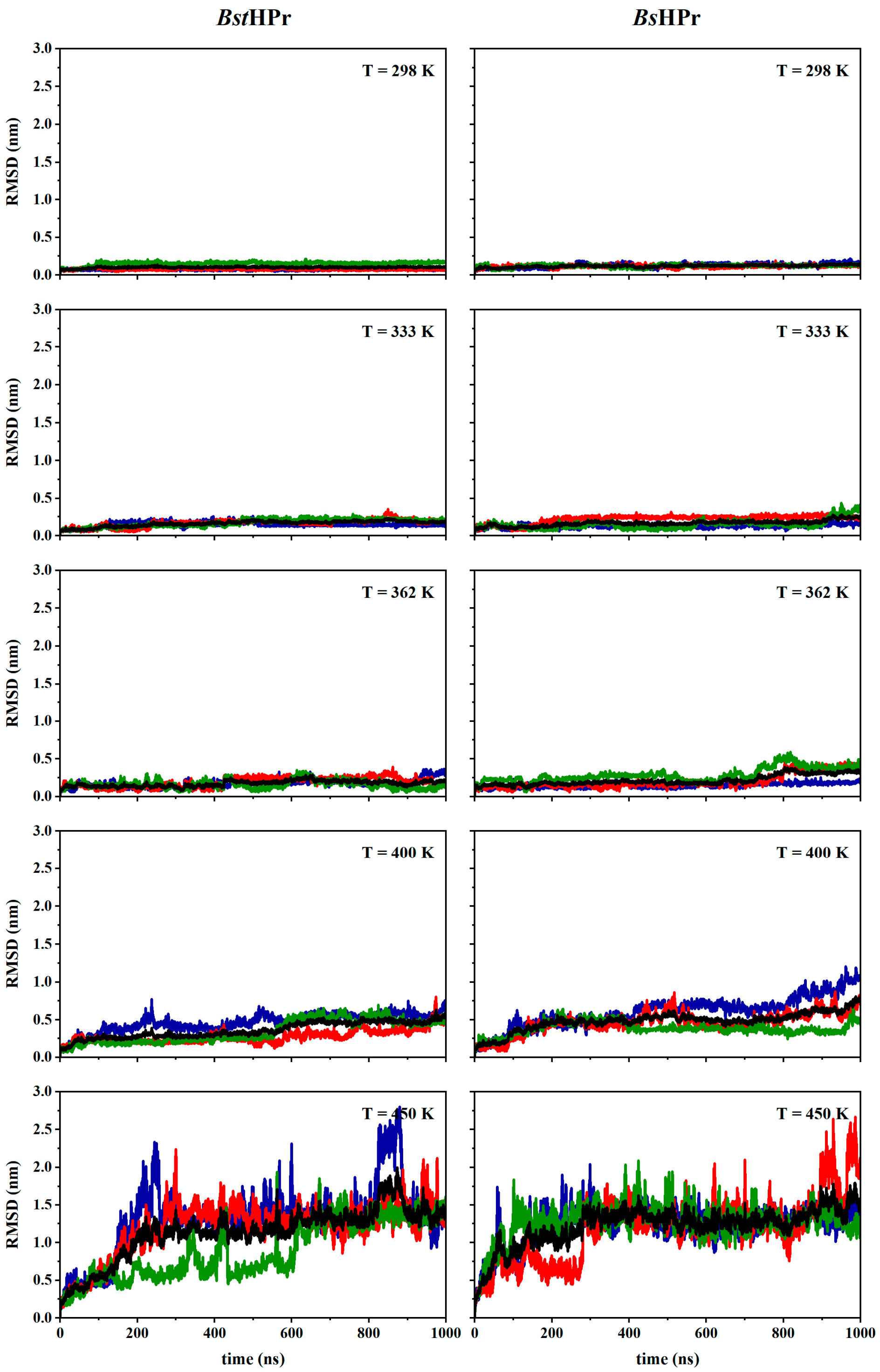

Figure S7. Behavior of the three independent trajectories of the RMSD for both proteins at temperatures of study. The trajectories of simulations 1, 2, and 3 are shown in blue, red, and green colored lines, respectively. The average values are shown in black lines.

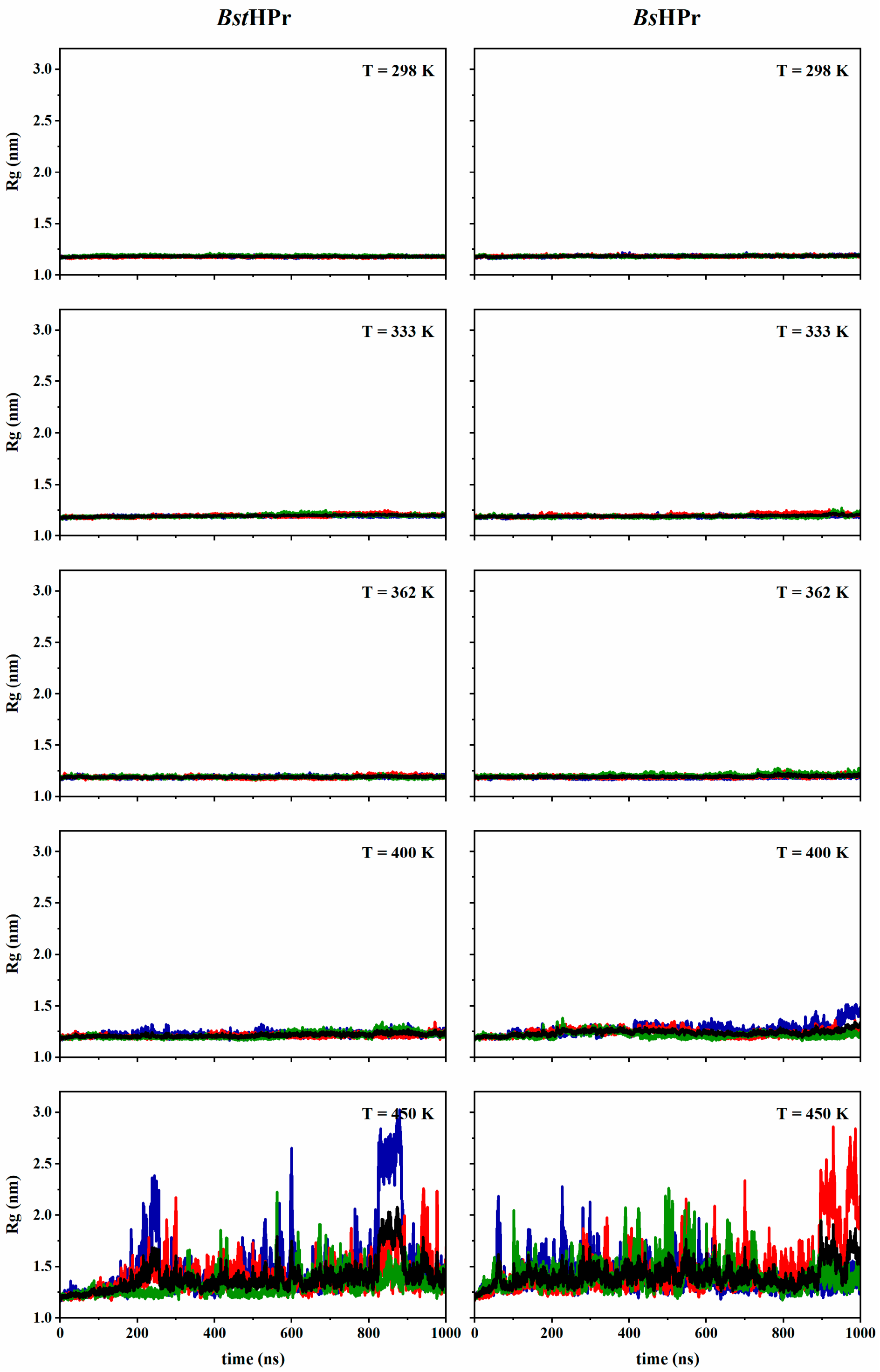

Figure S8. Behavior of the three independent trajectories of the Rg for both proteins at temperatures of study. The color codes are the same as in Figure S7.

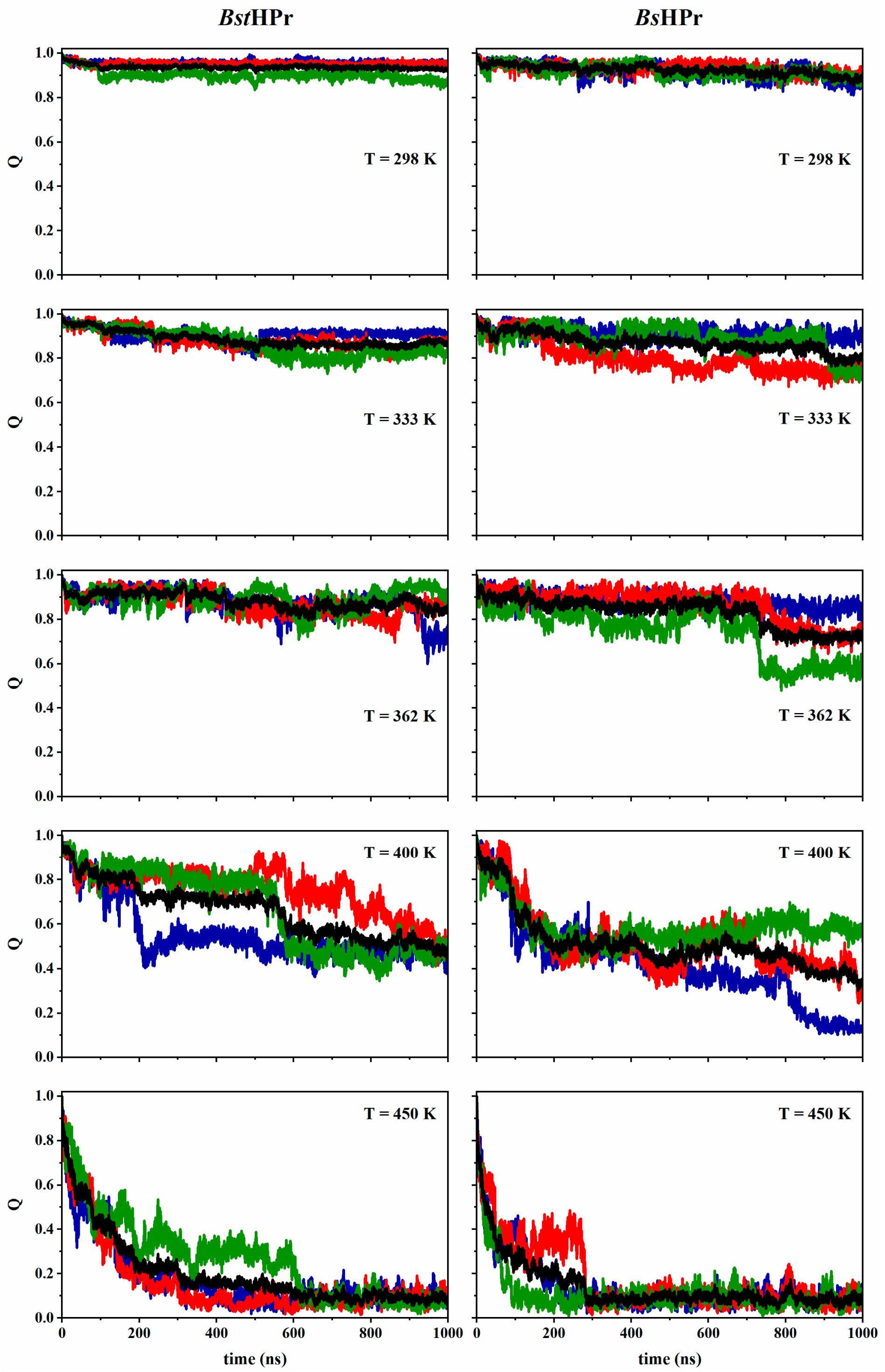

Figure S9. Behavior of the three independent trajectories of the fraction of native contacts for both proteins at temperatures of study. The color codes are the same as in Figure S7.

S6. Molecular Interaction Analyses

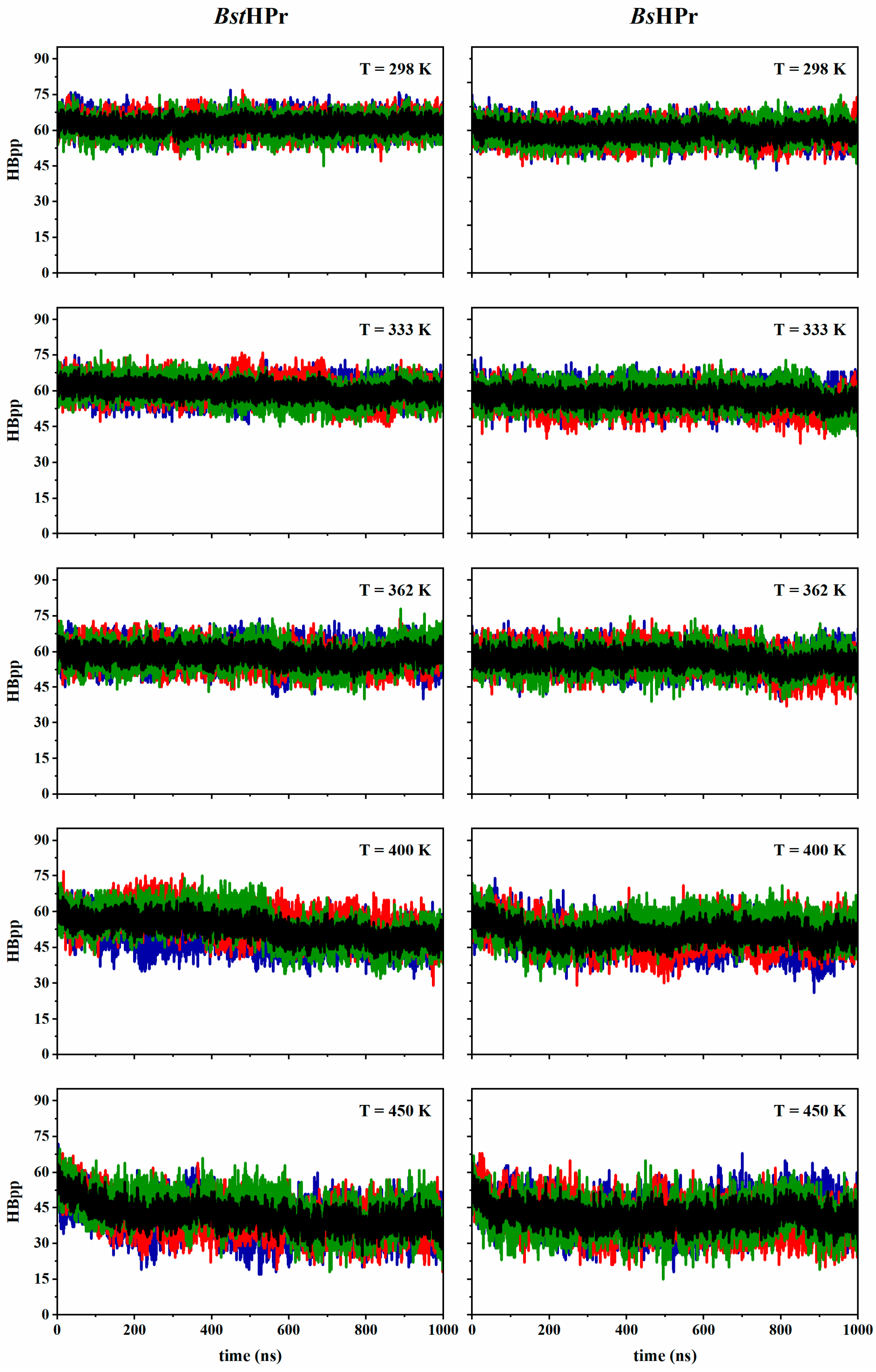

Figure S10. Behavior of the three independent trajectories of the protein–protein hydrogen bonds for both proteins at temperatures of study. The color codes are the same as in Figure S7.

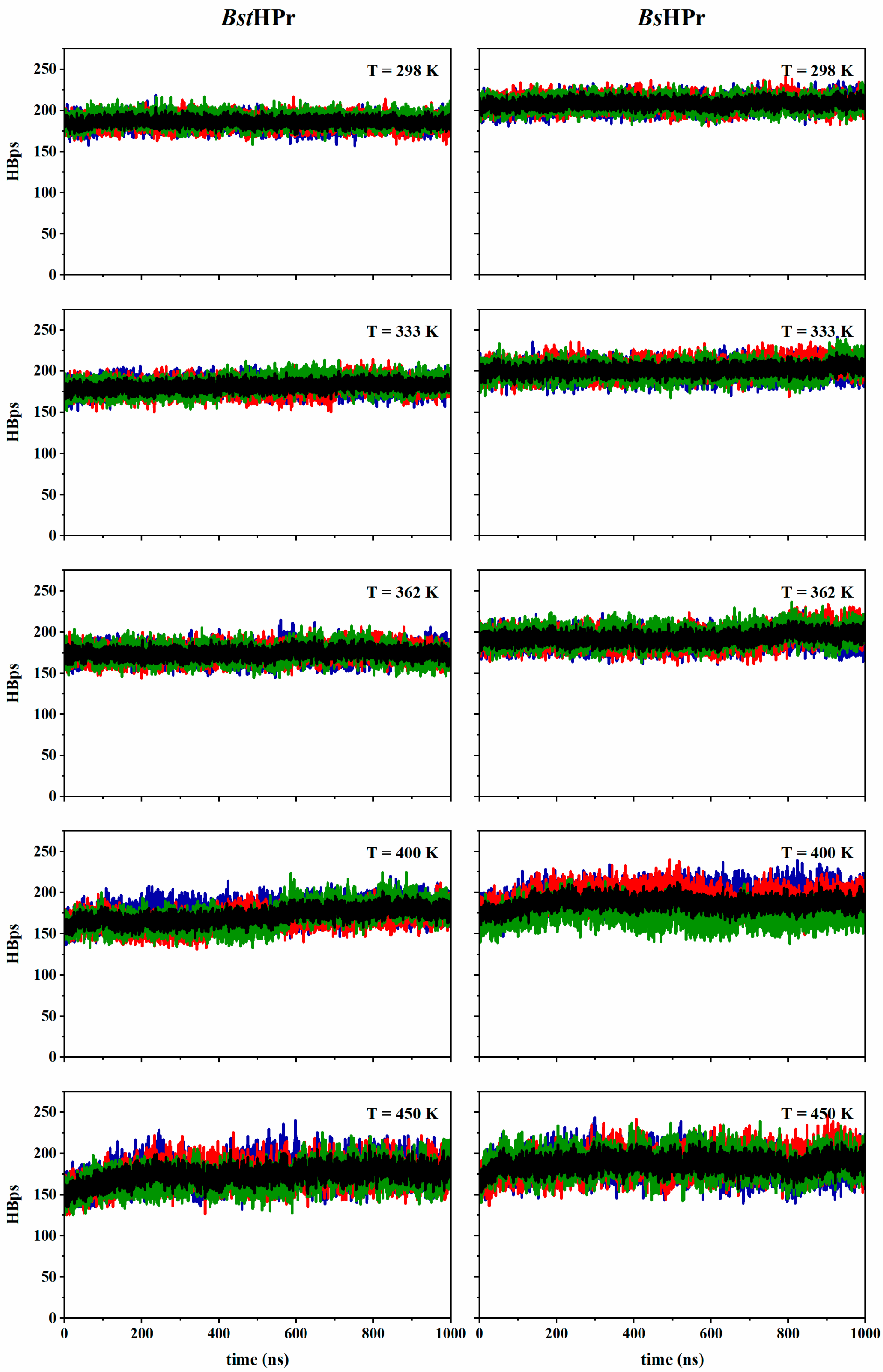

Figure S11. Behavior of the three independent trajectories of the protein–solvent hydrogen bonds for both proteins at temperatures of study. The color codes are the same as in Figure S7.

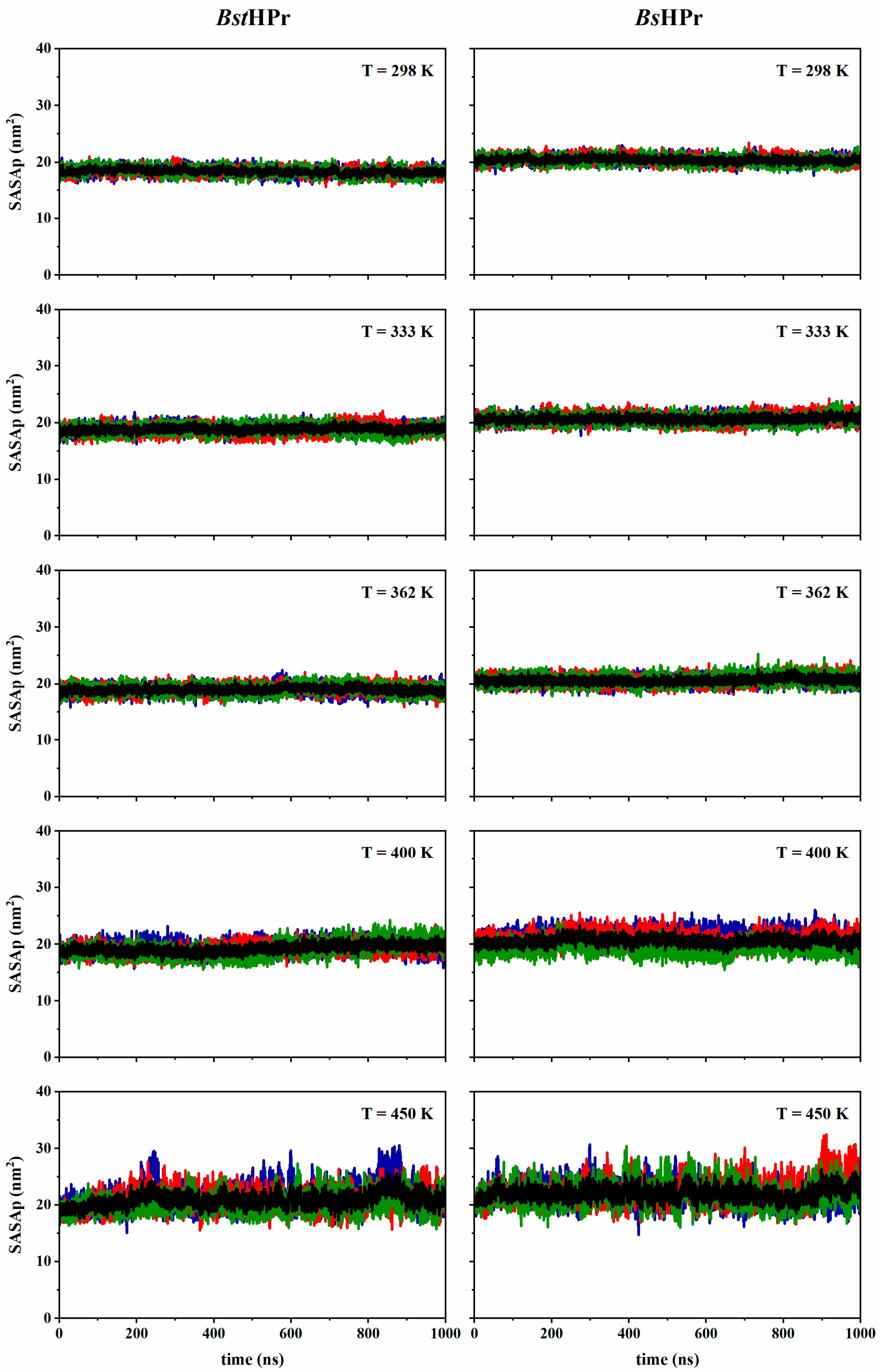

Figure S12. Behavior of the three independent trajectories of the solvent-accessible surface area for polar residues for both proteins at temperatures of study. The color codes are the same as in Figure S7.

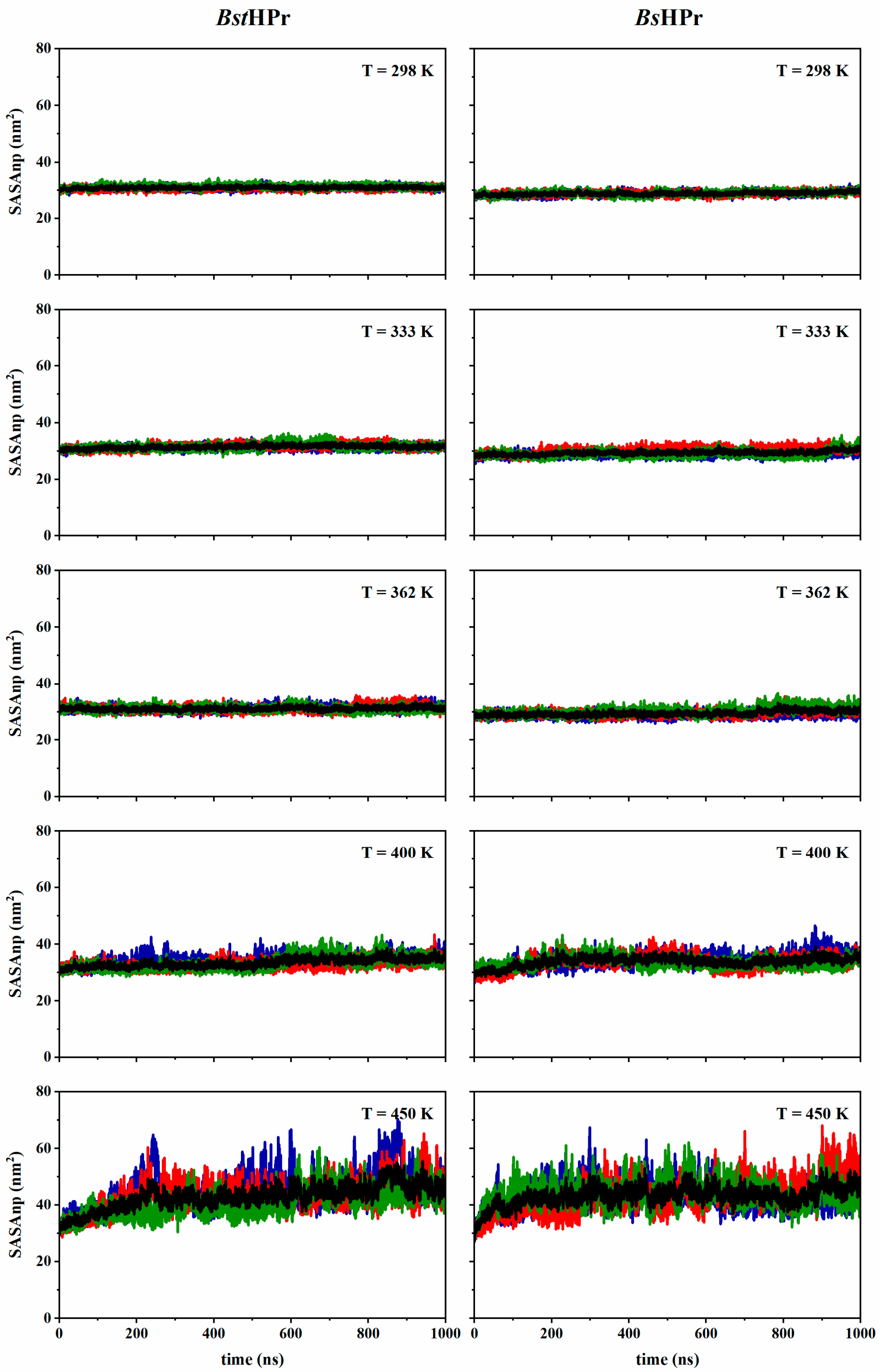

Figure S13. Behavior of the three independent trajectories of the solvent-accessible surface area for nonpolar residues for both proteins at temperatures of study. The color codes are the same as in Figure S7.

Table S1 shows the analyses of the ILV clusters for both proteins at 250, 500, 750, and 1000 ns for simulation 1 using the ProteinTools tool [10]. From this table, it can be got that the *Bs*HPr protein tends to form more ILV clusters as the temperature increases, indicating that its nonpolar residues become more exposed to the solvent compared with the *Bst*HPr protein.

From of the data described in Table S1, the ILV clusters were obtained for the conformations of the two proteins at 750 ns (Figure S14). While Figure S15 shows the behavior of the contacts per residue (C/R) of the main clusters. These contacts tend to decrease for the mesophilic protein as temperature increases, indicating that its structural instability increases due to thermal effects.

Table S1. ILV clusters for the *Bst*HPr and *Bs*HPr proteins at 250, 500, 750, and 1000 ns for the five temperatures studied.

t (ns)		Temperature (K)									
		298		333		362		400		450	
		***Bst*HPr**	***Bs*HPr**	***Bst*HPr**	***Bs*HPr**	***Bst*HPr**	***Bs*HPr**	***Bst*HPr**	***Bs*HPr**	***Bst*HPr**	***Bs*HPr**
**250**	NClus	1	1	1	1	1	1	1	1	2	2
	A	2911.3	2823.1	2789.4	2748.9	2906.9	2597.9	2135.6	2639.4	589.3/326	819.8/28.4
	NC	64	60	67	61	60	64	43	53	10/7	17/2
	C/R	3.8	3.3	3.7	3.6	3.5	3.6	3.3	3.5	2/1.8	2.1/1
	A/R	45.5	47.1	41.6	45.1	48.4	40.6	49.7	49.8	58.9/46.6	48.2/14.2
**500**	NClus	1	1	1	2	1	1	1	2	4	3
	A	2871.2	2984.9	2389.8	173.1/2415.3	2570.2	3116.8	2595.3	218.1/2517.5	33.7/142/84/546.5	799/37/50.5
	NC	66	60	59	4/53	59	66	58	4/55	2/4/2/10	19/2/2
	C/R	3.9	3.3	3.5	1.3/3.5	3.5	3.7	3.9	1.3/3.9	1/1.3/1/2	2.4/1/1
	A/R	43.5	49.7	40.5	43.3/45.6	43.6	47.2	44.7	54.5/45.8	16.8/35.6/42/54.7	42/18.8/25.3
**750**	NClus	1	1	1	1	1	1	1	2	2	2
	A	3283.4	2709.4	2295.2	2690.8	3195	2998.2	2246.9	1648.9/236.7	1510/127.2	816.5/69.1
	NC	67	69	58	59	69	71	56	43/4	28/2	22/2
	C/R	3.9	3.8	3.6	3.3	3.8	4.2	3.3	3.3/1.3	2.8/1	2.4/1
	A/R	49	39.3	39.6	45.6	46.3	42.2	40.1	38.3/59.2	53.9/63.6	37.1/34.6
**1000**	NClus	1	2	1	1	1	2	1	3	3	2
	A	3060.9	135.2/2506.2	2586.2	3105.3	2778.8	43/2000	1894.5	230/842/111	477/112.6/96	213.4/162.7
	NC	71	4/56	63	66	64	2/43	44	6/21/2	14/2/2	6/4
	C/R	4.2	1.3/3.7	3.9	3.7	4.3	1/2.9	3.7	1.5/2.3/1.0	2.3/1/1	1.5/1.3
	A/R	43.1	33.8/44.8	41.1	47	43.4	21.5/46.5	43.1	38.3/40/55.9	34.1/56.3/48	35.6/40.7

NClust = No. of Clusters; A = area (Å); NC = No. Contacts; C/R = Contacts/Residue; A/R = Area/Residue (Å)

***Bst*HPr** | ***Bs*HPr**

T = 298 K

*Bst*HPr

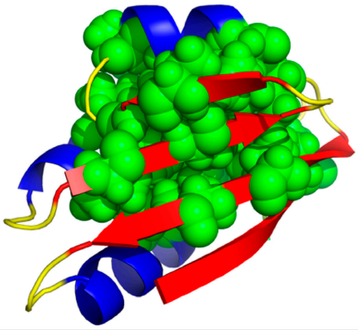

Cluster ID	Area (Å)	No. Contacts	Contacts/Residue	Area/Residue (Å)
0	3283.4	67	3.9	49.0

*Bs*HPr

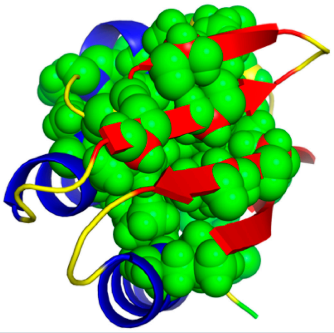

Cluster ID	Area (Å)	No. Contacts	Contacts/Residue	Area/Residue (Å)
0	2709.4	69	3.8	39.3

T = 333 K

*Bst*HPr

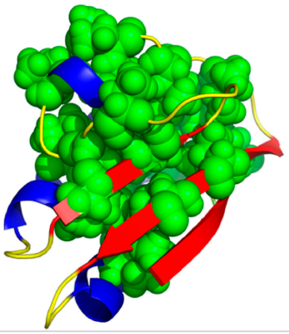

Cluster ID	Area (Å)	No. Contacts	Contacts/Residue	Area/Residue (Å)
0	2295.2	58	3.6	39.6

*Bs*HPr

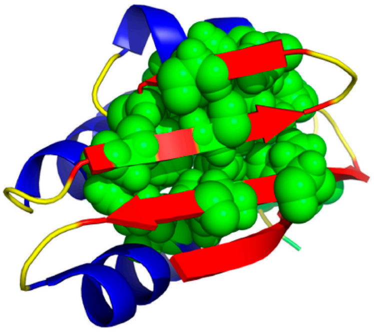

Cluster ID	Area (Å)	No. Contacts	Contacts/Residue	Area/Residue (Å)
0	2690.8	59	3.3	45.6

T = 362 K

*Bst*HPr

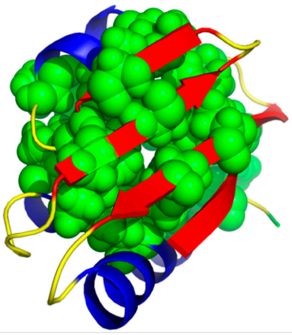

Cluster ID	Area (Å)	No. Contacts	Contacts/Residue	Area/Residue (Å)
0	3195.0	69	3.8	46.3

*Bs*HPr

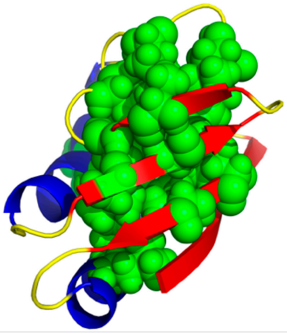

Cluster ID	Area (Å)	No. Contacts	Contacts/Residue	Area/Residue (Å)
0	2998.2	71	4.2	42.2

T = 400 K

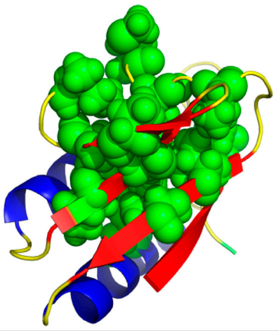

Cluster ID	Area (Å)	No. Contacts	Contacts/Residue	Area/Residue (Å)
0	2246.9	56	3.3	40.1

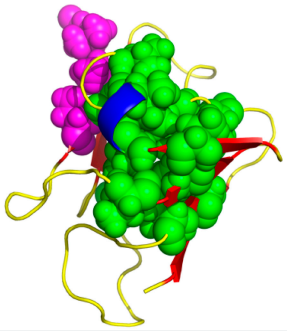

Cluster ID	Area (Å)	No. Contacts	Contacts/Residue	Area/Residue (Å)
0	1648.9	43	3.3	38.3
1	236.7	4	1.3	59.2

T = 450 K

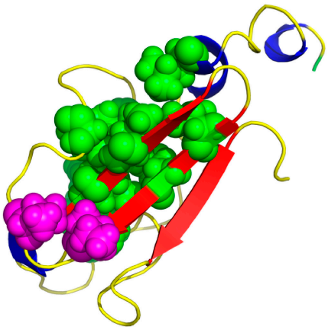

Cluster ID	Area (Å)	No. Contacts	Contacts/Residue	Area/Residue (Å)
0	1510.0	28	2.8	53.9
1	127.2	2	1.0	63.6

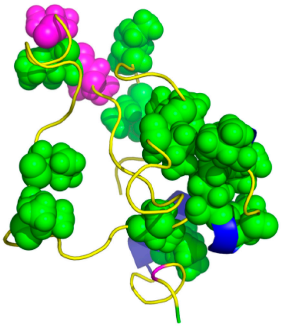

Cluster ID	Area (Å)	No. Contacts	Contacts/Residue	Area/Residue (Å)
0	816.5	22	2.4	37.1
1	69.1	2	1.0	34.6

Figure S14. ILV clusters for *Bst*HPr (**left panels**) and *Bs*HPr (**right panels**) at 750 ns at five temperatures analyzed. The red, blue, and yellow colors indicate the β-strand, α-helix, and random coil structures, respectively. ILV clusters are shown in green and magenta colors.

(a)

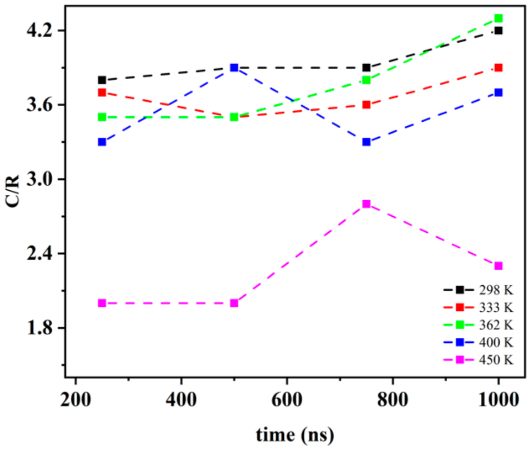

(b)

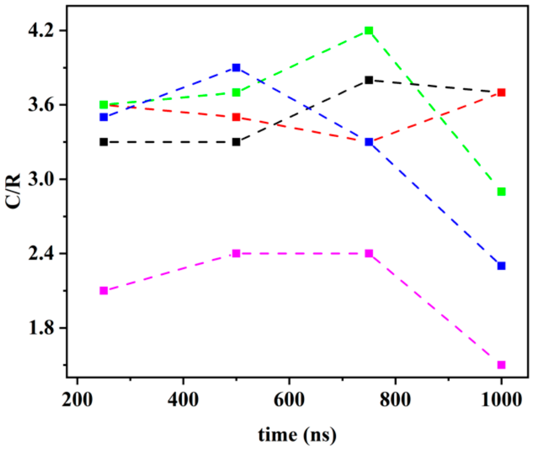

Figure S15. Contacts per residue of the main clusters at 250, 500, 750, and 1000 ns for: (**a**) *Bst*HPr and (**b**) *Bs*HPr proteins.

References

1. Kabsch, W.; Sander, C. Dictionary of Protein Secondary Structure: Pattern Recognition of Hydrogen-Bonded and Geometrical Features. *Biopolymers* **1983**, *22*, 2577−2637. https://doi.org/10.1002/bip.360221211
2. McGibbon, R.T.; Beauchamp, K.A.; Harrigan, M.P.; Klein, C.; Swails, J.M.; Hernańdez, C.X.; Schwantes, C.R.; Wang, L.P.; Lane, T.J.; Pande, V.S. MDTraj: A Modern Open Library for the Analysis of Molecular Dynamics Trajectories. *Biophys. J.* **2015**, *109*, 1528−1532. https://doi.org/10.1016/j.bpj.2015.08.015
3. Best, R.B.; Hummer, G.; Eaton, W.A. Native Contacts Determine Protein Folding Mechanisms in Atomistic Simulations. *Proc. Natl. Acad. Sci. U. S. A.* **2013**, *110*, 17874−17879. https://doi.org/10.1073/pnas.1311599110
4. Hubbard, R.E.; Haider, M.K. Hydrogen Bonds in Proteins: Role and Strength. In: Encyclopedia of Life Sciences (ELS). John Wiley & Sons, Ltd: Chichester 2010. https://doi.org/10.1002/9780470015902.a0003011.pub2
5. Mitternacht, S. FreeSASA: An Open Source C Library for Solvent Accessible Surface Area Calculations. *F1000Research* **2016**, *5*, 189. https://doi.org/10.12688/f1000research.7931.1
6. Lee, B.; Richards, F.M. The Interpretation of Protein Structures: Estimation of Static Accessibility. *J. Mol. Biol.* **1971**, *55*, 379−400. https://doi.org/10.1016/0022-2836(71)90324-X
7. Barlow, D. J.; Thornton, J. M. Ion-Pairs in Proteins. *J. Mol. Biol.* **1983**, *168*, 867−885. https://doi.org/10.1016/S0022-2836(83)80079-5
8. Baker, N.A.; Sept, D.; Joseph, S.; Holst, M.J.; McCammon, J.A. Electrostatics of Nanosystems: Application to Microtubules and the Ribosome. *Proc. Natl. Acad. Sci. U. S. A.* **2001**, *98*, 10037–10041. http://www.jstor.org/stable/3056478
9. Konecny, R.; Baker, N.A.; McCammon, J.A. iAPBS: A Programming Interface to the Adaptive Poisson–Boltzmann Solver. *Comput. Sci. Discov.* **2013**, *5*, 015005. https://doi.org/10.1088/1749-4699/5/1/015005
10. Ferruz, N.; Schmidt, S.; Höcker, B. ProteinTools: A Toolkit to Analyze Protein Structures. *Nucleic Acids Res.* **2021**, *49*, W559–W566. https://doi.org/10.1093/nar/gkab375
